# Supplementary material for: Fruit scent and observer colour vision shape food-selection strategies in wild capuchin monkeys
Source: Nat Commun. 2019 Jun 3;10:2407. doi: 10.1038/s41467-019-10250-9 (PMC6546703; doi:10.1038/s41467-019-10250-9)
Supplement: Supplementary file 1 — Supplementary Information [file 41467_2019_10250_MOESM1_ESM.pdf]

Supplementary Information

Fruit scent and observer colour vision shape food-selection strategies in wild capuchin monkeys

Amanda D. Melin[1,2*], Omer Nevo[3], Mika Shirasu[4,5], Rachel E. Williamson[1], Eva Garrett[6], Mizuki Endo[7], Kodama Sakurai[7], Yuka Matsushita[7], Kazushige Touhara[4,5], Shoji Kawamura[7*]

[1]Department of Anthropology and Archaeology, University of Calgary, Calgary, AB T2N 1N4, Canada. [2]Department of Medical Genetics and Alberta Children's Hospital Research Institute, Cumming School of Medicine, University of Calgary, Calgary, AB T2N 4N1, Canada. [3]Institute of Evolutionary Ecology and Conservation Genomics, University of Ulm, Albert-Einstein-Allee 11, 89081 Ulm, Germany. [4]Department of Applied Biological Chemistry, Graduate School of Agricultural and Life Sciences, The University of Tokyo, Bunkyo-ku, Tokyo 113-8657, Japan. [5]ERATO Touhara Chemosensory Signal Project, JST, The University of Tokyo, Tokyo 113-8657, Japan. [6]Department of Anthropology, Boston University, Boston, MA. [7]Department of Integrated Biosciences, Graduate School of Frontier Sciences, The University of Tokyo, Kashiwa, Chiba 277-8562, Japan. *e-mail: amanda.melin@ucalgary.ca and kawamura@edu.k.u-tokyo.ac.jp.

This PDF file includes:

Supplementary Figures 1, 2, 3

Supplementary Tables 1, 2, 3

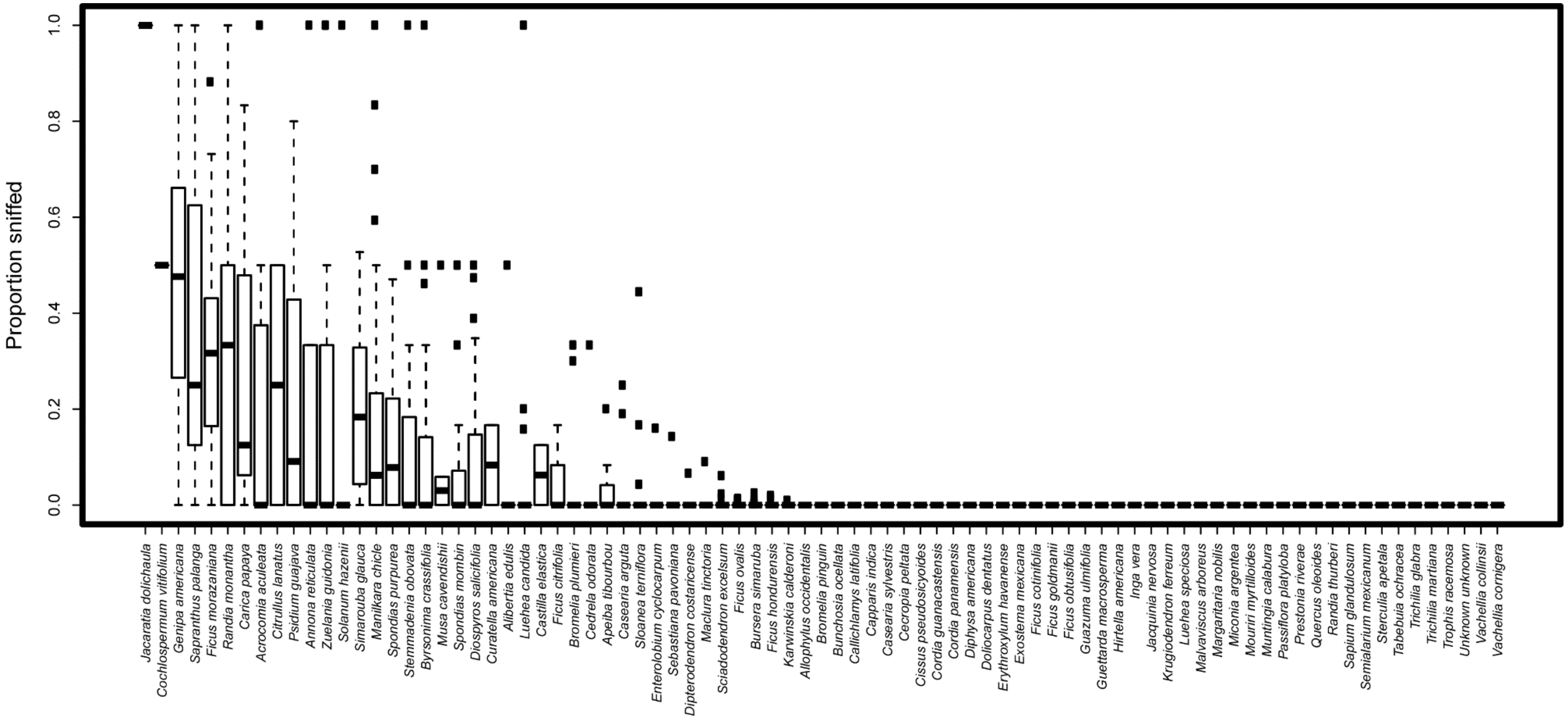

Supplementary Figure 1 | Variation in fruit sniffing behaviour by white-faced capuchins for 83 plant species. Box plots of fruit investigation sequences (N = 46 709) by individual white-faced capuchin monkeys that included at least 1 sniffing event. Medians (bar) are plotted along with upper and lower quartiles (box perimeters), and whiskers stretching to the first data point within 1.5 inter-quartile ranges of the box. Points beyond the whiskers are plotted as individual symbols. Source data are provided as a Source Data file.

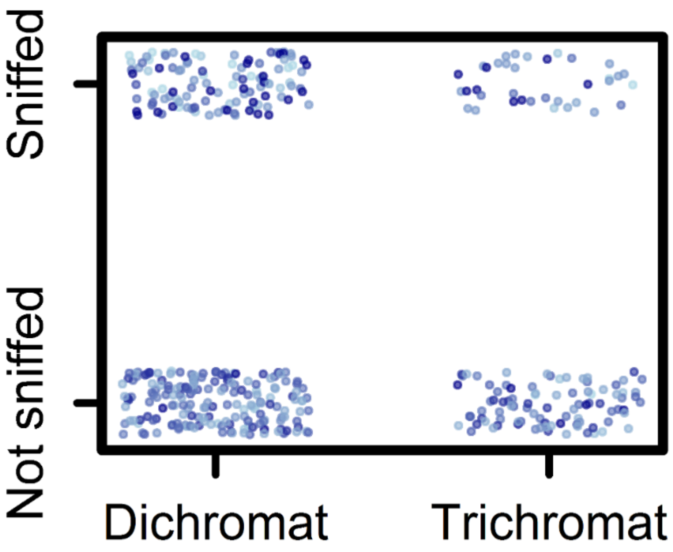

Supplementary Figure 2 | Sniffing behaviour and primate colour vision type plotted with blue-yellow contrast between unripe and ripe fruits. Each dot represents an individual monkey-plant species combination. Data points were binomially classified as to whether that monkey was ever observed sniffing the fruits of that plant species and are vertically and horizontally jittered in each quadrant. Darker blue dots represent a larger blue-yellow chromatic contrast between ripe and unripe fruits in the visual space of a trichromatic capuchin monkey. Dichromatic monkeys sniffed fruits significantly more often than trichromatic monkeys did, regardless of blue-yellow contrast; glmm: $p < 0.01$. Source data are provided as a Source Data file.

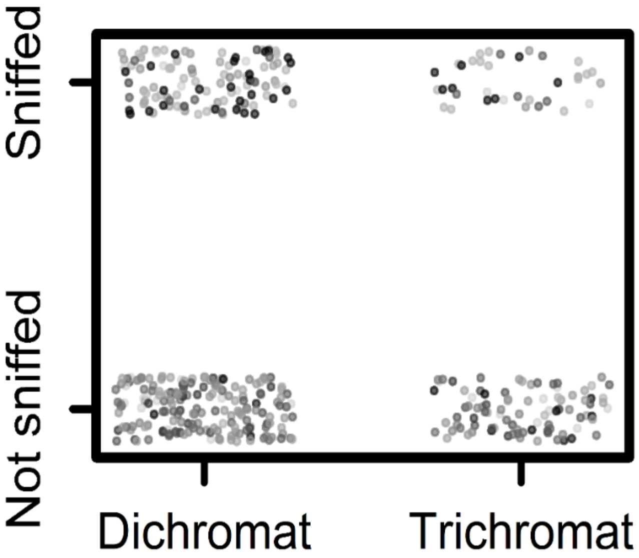

Supplementary Figure 3 | Sniffing behaviour and primate colour vision type plotted with luminance contrast between unripe and ripe fruits. Each dot represents an individual monkey-plant species combination. Data points were binomially classified as to whether that monkey was ever observed sniffing the fruits of that plant species and are vertically and horizontally jittered in each quadrant. Darker dots represent a larger luminance contrast between ripe and unripe fruits in the visual space of a trichromatic capuchin monkey. Dichromatic monkeys sniffed fruits significantly more often than trichromatic monkeys did, regardless of luminance contrast; glmm: $p < 0.01$. Source data are provided as a Source Data file.

Supplementary Table 1 | Results of generalized linear mixed model – females only. Coefficients and p-values of all fixed effects. Males were excluded to test the effects of colour vision phenotype while removing any influence of sex (Dichromatic females, n = 17; Trichromatic females, n=24). P values in bold are statistically significant at $p < 0.05$.

	**Estimate**	**Std. Error**	**z value**	**p value**
Intercept	-0.07	1.74	-0.04	0.97
Colour vision type	-1.54	0.46	-3.32	**<0.001**
Scent increase ratio (log transformed)	0.99	0.51	1.39	**0.05**
Chemical dissimilarity	-4.53	3.2	-1.42	0.16

Supplementary Table 2 | Results of generalized linear mixed model – dichromats only. Coefficients and p-values of all fixed effects. Trichromats were excluded to test the effects of sex while removing influence of colour vision phenotype. (Dichromatic males n = 34, Dichromatic females, n=17). P values in bold are statistically significant at $p < 0.05$.

	**Estimate**	**Std. Error**	**z value**	**p value**
Intercept	-0.14	1.79	-0.08	0.94
Sex	-0.83	0.4	-2.09	**0.04**
Scent increase ratio (log transformed)	0.96	0.5	1.93	0.05
Chemical dissimilarity	-3.99	3.25	-1.23	0.22

Supplementary Table 3 | Fruit sampling details. Number of fruits sampled for scent measurement by plant species in each sampling season.

Plant species	Sampling Season	Number collected per ripeness stage
Byrsonima crassifolia	1	5
Cordia guanacastensis	1	20
Cordia panamensis	1	10
Diospyros salicifolia	2	5
Ficus morazaniana	2	5
Genipa americana	1	1
Guettarda macrosperma	1	5
Jacquinia nervosa	1	4
Karwinskia calderoni	2	5
Maclura tinctoria	1	5
Malvaviscus arboreus	1	7
Psidium guajava	1	1
Randia thurberi	1	5
Sciadodendron excelsum	2	5
Simarouba glauca	2	5
Spondias mombin	1	5
Stemmadenia obovata	1	1
Vachellia collinsii	1	6
