# Supplementary material for: Fruit scent and observer colour vision shape food-selection strategies in wild capuchin monkeys
Source: Nat Commun. 2019 Jun 3;10:2407. doi: 10.1038/s41467-019-10250-9 (PMC6546703; doi:10.1038/s41467-019-10250-9)
Supplement: Supplementary file 3 — Reporting Summary [file 41467_2019_10250_MOESM3_ESM.pdf]

nature research

Corresponding author(s): Amanda D Melin

☐ Initial submission ☐ Revised version ☒ Final submission

Reporting Summary

Nature Research wishes to improve the reproducibility of the work that we publish. This form provides structure for consistency and transparency in reporting. For further information on Nature Research policies, see Authors & Referees and the Editorial Policy Checklist.

Please do not complete any field with "not applicable" or n/a. Refer to the help text for what text to use if an item is not relevant to your study.
For final submission: please carefully check your responses for accuracy; you will not be able to make changes later.

Statistical parameters

When statistical analyses are reported, confirm that the following items are present in the relevant location (e.g. figure legend, table legend, main text, or Methods section).

n/a	Confirmed	
☐	☒	The exact sample size (n) for each experimental group/condition, given as a discrete number and unit of measurement
☐	☒	An indication of whether measurements were taken from distinct samples or whether the same sample was measured repeatedly
☐	☒	The statistical test(s) used AND whether they are one- or two-sided<br>*Only common tests should be described solely by name; describe more complex techniques in the Methods section.*
☐	☒	A description of all covariates tested
☐	☒	A description of any assumptions or corrections, such as tests of normality and adjustment for multiple comparisons
☐	☒	A full description of the statistics including central tendency (e.g. means) or other basic estimates (e.g. regression coefficient) AND variation (e.g. standard deviation) or associated estimates of uncertainty (e.g. confidence intervals)
☐	☒	For null hypothesis testing, the test statistic (e.g. F, t, r) with confidence intervals, effect sizes, degrees of freedom and P value nc<br>*Give P values as exact values whenever suitable.*
☒	☐	For Bayesian analysis, information on the choice of priors and Markov chain Monte Carlo settings
☒	☐	For hierarchical and complex designs, identification of the appropriate level for tests and full reporting of outcomes
☒	☐	Estimates of effect sizes (e.g. Cohen's d, Pearson's r), indicating how they were calculated
☐	☒	Clearly defined error bars<br>*State explicitly what error bars represent (e.g. SD, SE, CI)*

Our web collection on statistics for biologists may be useful.

Software and code

Policy information about availability of computer code

Data collection	We collected behavioural data onto hand-held PSION workabout computers (Noldus), running the program Behavior, which records the text entered, along with a date-time stamp.<br><br>We recorded spectral reflectance data with a portable USB4000 spectrophotometer (Ocean Optics) and SpectraSuite software
Data analysis	We used MATLAB R2015a to analyze the spectral reflectance data from fruits (see supplementary note 2)<br><br>We used R to analyze the behavioral dataset. Our code is posted on GitHub https://github.com/omernevo/Variation-in-capuchin-sniffing-project.<br><br>We analyzed the GC-MS data using Analyzer Pro (SpectralWorks, Cheshire, UK). Individual peaks were tentatively identified using the National Institute of Standards and Technology library database (NIST 14) using a greater than 85% confidence criterion.

For manuscripts utilizing custom algorithms or software that are central to the research but not yet described in published literature, software must be made available to editors/reviewers upon request. We strongly encourage code deposition in a community repository (e.g. GitHub). See the Nature Research guidelines for submitting code & software for further information.

Data

Policy information about availability of data

All manuscripts must include a data availability statement. This statement should provide the following information, where applicable:

- Accession codes, unique identifiers, or web links for publicly available datasets
- A list of figures that have associated raw data
- A description of any restrictions on data availability

Primate foraging data, chromatic and luminance distance data between ripe and unripe fruits, and chemical distance data between ripe and unripe fruits are available in the Source Data File and published open access on the Zenodo repository, DOI: 10.5281/zenodo.2634368. Behavioural data for all fruit species were used to generate Fig S1. Data for fruit species accompanied by fruit trait data were used to generate Figs 1 - 4 and Supplementary Figs 2-3.

Field-specific reporting

Please select the best fit for your research. If you are not sure, read the appropriate sections before making your selection.

☐ Life sciences ☒ Behavioural & social sciences ☐ Ecological, evolutionary & environmental sciences

For a reference copy of the document with all sections, see nature.com/authors/policies/ReportingSummary-flat.pdf

Behavioural & social sciences study design

All studies must disclose on these points even when the disclosure is negative.

Study description	We integrate behavioral study of wild primates with study of fruit properties to investigate the sensory ecology of food selection. Data collected in this study are quantitative (assessment of sniffing behavior, assessment of fruit color conspicuity, analysis of volatile organic compounds (VOCs). Color vision phenotypes (based on analysis of opsin genotypes of study monkeys) are published elsewhere (Melin et al. 2017. PNAS 114:10402-10407)
Research sample	We studied 75 individual white-faced capuchin monkeys from 4 social groups, 51 dichromats (red-green colorblind males and females) and 24 trichromats (females with colour vision similar to normal human color vision). Capuchins in Sector Santa Rosa were selected because they are individually known, have color vision polymorphism, a well characterized diet, and are wild but habituated. The monkeys forage on natural foods (i.e. are not provisioned) and viewing conditions in the dry forest are much better than in nearly all other Neotropical forests.<br><br>The age-sex composition and color vision type of these groups are representative of the broader population.<br><br>We recorded sensory behaviour while feeding on 83 plant species. We conducted fruit trait analysis (chemical composition of scent; color) for a subset of 18 plant species.
Sampling strategy	We conducted focal animal sampling for up to 10 minutes duration. We were flexible with the duration of the focal follow to accommodate challenges in maintaining clear views of face and hands at all times, and to sample as many monkeys as possible in the same tree to control for ecological conditions. We recorded detailed food investigation sequences and noted whether the fruit was sniffed or not before being eaten or rejected. We discarded sequences for which we could not clearly see the handing of the fruit, including the final outcome. A two-observer system (one caller, one recorder) was used so that the observer did not look away from the focal monkey, even fleetingly, to record a behaviour. When the observer's view of the monkey was partially obstructed, "out of sight" was coded until a clear view was again available.<br><br>We only included fruit species in the behavioral analysis for which we had behavioural data for both dichromatic and trichromatic monkeys. In total we used foraging data for fruits of 83 species (46 709 fruit investigation events). All data will be available on Dryad.<br><br>Data on fruit colour and scent were collected for a subset, 18 species, of the fruit species we observed capuchins to eat. We selected fruits semi-randomly based on availability, including those that we observed capuchins to sniff frequently as well as fruit species they did not often sniff. We made sure to include fruit species that did undergo a conspicuous color change during ripening, as well as fruit species that did not. All data will be available on Dryad.
Data collection	We recorded behavioural data on a hand-held PSION workabout computer. A two-observer system (one caller, one recorder) was used so that the observer did not look away from the focal monkey, even fleetingly, to record a behaviour. When the observer's view of the monkey was partially obstructed, "out of sight" was coded until a clear view was again available. At any one time a maximum of 4 field researchers were following one group of monkeys.<br><br>Fruits were collected in the field, using pruning poles when needed, and transported to the field stations. The color and odor measurements began within 3-4 hours of collection. Colour was measured using a portable spectrophotometer (USB4000, OceanOptics) and LS-1 light source. Scent was sampled using dynamic headpsace technique and analyzed using gas-chromatography and mass-spectrometry (for more details see Methods and supplementary information 1, 3-4).

	At the time of study, A.D.M. was aware that males would be dichromatic and knew the genotypes of five out of 24 adult females from a previous study (41); however, color vision types were intentionally not revealed to five assistants collecting behavioral data.
Timing	We collected behavioural data on primates over 25 months nearly equally split between the rainy (typically mid-May through mid November annually), and dry seasons: May – July 2004, January – May 2005, January–May 2007, September 2007–January 2008, and May–September 2008, February 2009, June 2009 and August 2009.<br><br>We collected data on fruit colors and odors over two field seasons: 1) July - August 2012; and 2) December 2015 - June 2016 (for details see Supplementary Note 1).
Data exclusions	Data from unidentified monkeys, or from monkeys whose color-vision phenotype was unknown, were excluded from analyses.
Non-participation	Several individual monkeys disappeared from the study groups between seasons, due to death or emigration.
Randomization	Individual ID was nested within social group membership in statistical models to control for repeated measures of the same monkeys foraging on different fruits.

Reporting for specific materials, systems and methods

We require information from authors about some types of materials, experimental systems and methods used in many studies. Here, indicate whether each material, system or method listed is relevant to your study. If you are not sure if a list item applies to your research, read the appropriate section before selecting a response.

Materials & experimental systems

n/a	Involved in the study	
☒	☐	Unique biological materials
☒	☐	Antibodies
☒	☐	Eukaryotic cell lines
☒	☐	Palaeontology
☐	☒	Animals and other organisms
☒	☐	Human research participants

Methods

n/a	Involved in the study	
☒	☐	ChIP-seq
☒	☐	Flow cytometry
☒	☐	MRI-based neuroimaging

Animals and other organisms

Policy information about studies involving animals; ARRIVE guidelines recommended for reporting animal research

Laboratory animals	*For laboratory animals, report species, strain, sex and age OR state that the study did not involve laboratory animals.*
Wild animals	We followed and observed 4 social groups of wild monkeys. No monkeys were handled or injured during this study.
Field-collected samples	Genetic data (previously reported) came from fecal DNA collected non-invasively.

This checklist template is licensed under a Creative Commons Attribution 4.0 International License, which permits use, sharing, adaptation, distribution and reproduction in any medium or format, as long as you give appropriate credit to the original author(s) and the source, provide a link to the Creative Commons license, and indicate if changes were made. The images or other third party material in this article are included in the article's Creative Commons license, unless indicated otherwise in a credit line to the material. If material is not included in the article's Creative Commons license and your intended use is not permitted by statutory regulation or exceeds the permitted use, you will need to obtain permission directly from the copyright holder. To view a copy of this license, visit http://creativecommons.org/licenses/by/4.0/
