# Supplementary material for: Fruit scent and observer colour vision shape food-selection strategies in wild capuchin monkeys
Source: Nat Commun. 2019 Jun 3;10:2407. doi: 10.1038/s41467-019-10250-9 (PMC6546703; doi:10.1038/s41467-019-10250-9)
Supplement: Supplementary file 4 — Description of Additional Supplementary Files [file 41467_2019_10250_MOESM4_ESM.pdf]

Description of Additional Supplementary Files

Supplementary Data 1

Summary of study individuals and foraging data. We provide the sex, social group, age class, and color vision type for each capuchin monkey in our study. Values represent the number of fruit investigations per individual for plant species included in the odour analyses.
